# Two cases of von Willebrand disease type 3 in consanguineous Chinese families

**DOI:** 10.1002/mgg3.1075

**Published:** 2019-12-02

**Authors:** Xiong Wang, Ning Tang, Yanjun Lu, Qun Hu, Dengju Li

**Affiliations:** ^1^ Department of Laboratory Medicine Tongji Hospital Tongji Medical College Huazhong University of Science and Technology Wuhan China; ^2^ Department of Pediatrics Tongji Hospital Tongji Medical College Huazhong University of Science and Technology Wuhan China; ^3^ Department of Hematology Tongji Hospital Tongji Medical College Huazhong University of Science and Technology Wuhan China

**Keywords:** autosomal recessive, consanguineous marriage, *F8*, von Willebrand disease, *VWF*

## Abstract

**Background:**

von Willebrand disease (VWD) is the most common inherited bleeding disorder caused by defective or deficient von Willebrand factor (VWF). VWD type 3 is inherited in autosomal recessive manner. We described clinical and molecular features of VWD type 3 in two consanguineous marriage families.

**Methods:**

Peripheral blood was collected, PT, APTT, FVIII:C, VWF:RCo, VWF:Ag were measured. A targeted next‐generation sequencing panel covering *F8*, *F9*, and *VWF* genes was applied followed by Sanger sequencing.

**Results:**

Both families had a baby die in their first year due to bleeding disorders. A 23‐year‐old female patient from family A suffered menorrhagia, and another 30‐year‐old male patient from family B was characterized with hematoma in the lower extremity. Both patients showed severely decreased FVIII:C, VWF:Ag. Recurrent homozygous *VWF* c.4696C>T (p.Arg1566Ter) nonsense mutation was identified in the female patient, and novel homozygous *VWF* c.6450C>A (p.Cys2150Ter) nonsense mutation was identified the male patient. Heterozygotes in family members showed mild/moderate decrease in VWF:Ag or VWF:RCo.

**Conclusions:**

We identified VWD type 3 in two consanguineous marriage families, and our work further strengthen the risk of delivering disorders inherited in AR manner in populations with frequent consanguineous partnerships.

## Graphical abstract

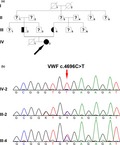

## 1 INTRODUCTION

von Willebrand disease (VWD) is the most common congenital bleeding disorder caused by quantitative (types 1 and 3) or qualitative (type 2) deficiency of von Willebrand factor (VWF), and its clinical manifestations include mild to severe mucocutaneous bleeding (Swami & Kaur, 2017). The prevalence of VWD is estimated at 1% based on epidemiological studies, but is lower according to studies of patients presenting for clinical assessment/treatment (Favaloro, 2011).

VWD is usually caused by mutations of the *VWF* gene located on 12p13.3. The VWF protein plays essential roles in the interaction of platelets with vessel injury via binding GPIbα through A1 domain and binding vascular collagens through A1 and A3 domains (Pareti, Niiya, Mcpherson, & Ruggeri, 1987). Another role of VWF is to protect FVIII in the transport and stabilization via binding FVIII trhough D'D3 domain (Foster, Fulcher, Marti, Titani, & Zimmerman, 1987). VWD is sub‐grouped into type 1, 2, 3. VWD type 1 is caused by partial quantitative deficiency of VWF, and type 3 is caused by complete quantitative deficiency of VWF, whereas type 2 is caused by qualitative deficiency of defective VWF. VWD type 2 can be further classified into 2A, 2B, 2M, and 2N. The characterization of VWD type 2 is based on functional defects that lead to impaired activity and is accordingly divided into these four secondary categories.

The diagnosis of VWD is established based on mucocutaneous bleeding, results of hemostasis tests, and a positive family history. VWD types 1, 2A, 2B, 2M are mainly inherited in autosomal dominant (AD) manner. VWD types 2N and 3 are inherited in autosomal recessive (AR) manner. Incomplete penetrance and variable expressivity of VWD makes the diagnosis challenging (Baronciani, Goodeve, & Peyvandi, 2017). Molecular diagnosis of VWD is helpful for VWD classification and genetic counselling for evaluating the risk of recurrence. Next‐generation sequencing (NGS) has been widely used in the detection of bleeding disorders including VWD (Bastida et al., 2017; Borras et al., 2017; Fidalgo et al., 2016). The rate of VWD type 3 was elevated in populations with frequent consanguineous partnerships.

In this study, we described the clinical and molecular features of VWD type 3 in two consanguineous marriage families.

## 2 MATERIALS AND METHODS

### 2.1 Ethical compliance

This study was approved by the Ethics Committee of Tongji Hospital, Tongji Medical College, Huazhong University of Science and Technology. The study was performed in accordance with the Declaration of Helsinki and written informed consent for molecular study and publication was obtained from all participants.

### 2.2 Patients and laboratory tests

Patients and their family members were included. FVIII:C was measured by the one‐stage coagulative method (Diagnostic Stago) on Stago STA‐R automated coagulation factor analyzer. VWF antigen (VWF:Ag) was detected by turbidimetric inhibition immuno assay on ACL TOP TM Hemostasis Testing System (Instrumentation Laboratory). VWF ristocetin cofactor (VWF:RCo) was performed by washed platelet aggregometry on a AggRAM platelet aggregation analyzer (Helena Laboratory). PBMCs were collected and genome DNA was extracted. Laboratory tests for the two patients were shown in Table 1.

**Table 1:** Laboratory tests for the two patients

Test	Results patient A	Results patient B	Reference interval	Unit
PT	15.9 (↑)	13.6	11.5–14.5	s
APTT	62.7 (↑)	72.0 (↑)	29.0–42.0	s
TT	14.9	14.9	14.0–19.0	s
FVIII:C	1.3 (↓)	1.2 (↓)	60–150	%
VWF:Ag	3.6 (↓)	0.5 (↓)	42.0–140.8 (O blood group) 66.1–176.3 (A, B, or AB blood group)	%

Abbreviations: APTT, activated partial thromboplastin time; PT, prothrombin time; TT, thrombin time.

### 2.3 NGS and Sanger sequencing

NGS was performed targeting all exons and intronic flanking regions of the *F8* (OMIM: * 300841, NM_000132.3), *F9* (OMIM: * 300746, NM_000133.3), and *VWF* (OMIM: * 613160, NM_000552.4) genes. Some exons of *VWF* gene could not be discriminated from its highly homologous pseudogene, and these exons were sequenced by Sanger sequencing.

DNA libraries were prepared by the Ampliseq Library Preparation Kit (Thermo Fisher), and purified with AMPure XP beads (Beckman Coulter), quantified using the KAPA Library Quantification Kit (KAPA Biosystems), and were further pooled together in equal amount. The pooled library was amplified by emulsion PCR on OT2 machine, and analyzed by Semiconductor sequencing on the Ion Torrent PGM using the Ion 316 chip.

### 2.4 Bioinformatic analysis

Data were aligned with the human hg19 reference genome sequence. Coverage analysis and variant caller plugins were performed according to the default parameters on the Ion Torrent Server version 4.4.2. Identified variants were annotated with the Ion Reporter version 5.2 according to the variant nomenclature recommendation by the HGVS. Regions covered less than 20 folds were visually verified with IGV v2.3.8 (Broad Institute). Annotated variants were searched in several databases, including 1000G, dbSNP, ClinVar, gnomAD, ExAC, and HGMD databases. Functional prediction was performed using PolyPhen‐2, SIFT, MutationTaster, and InterVar.

## 3 RESULTS

### 3.1 Family A

**Figure 1:**
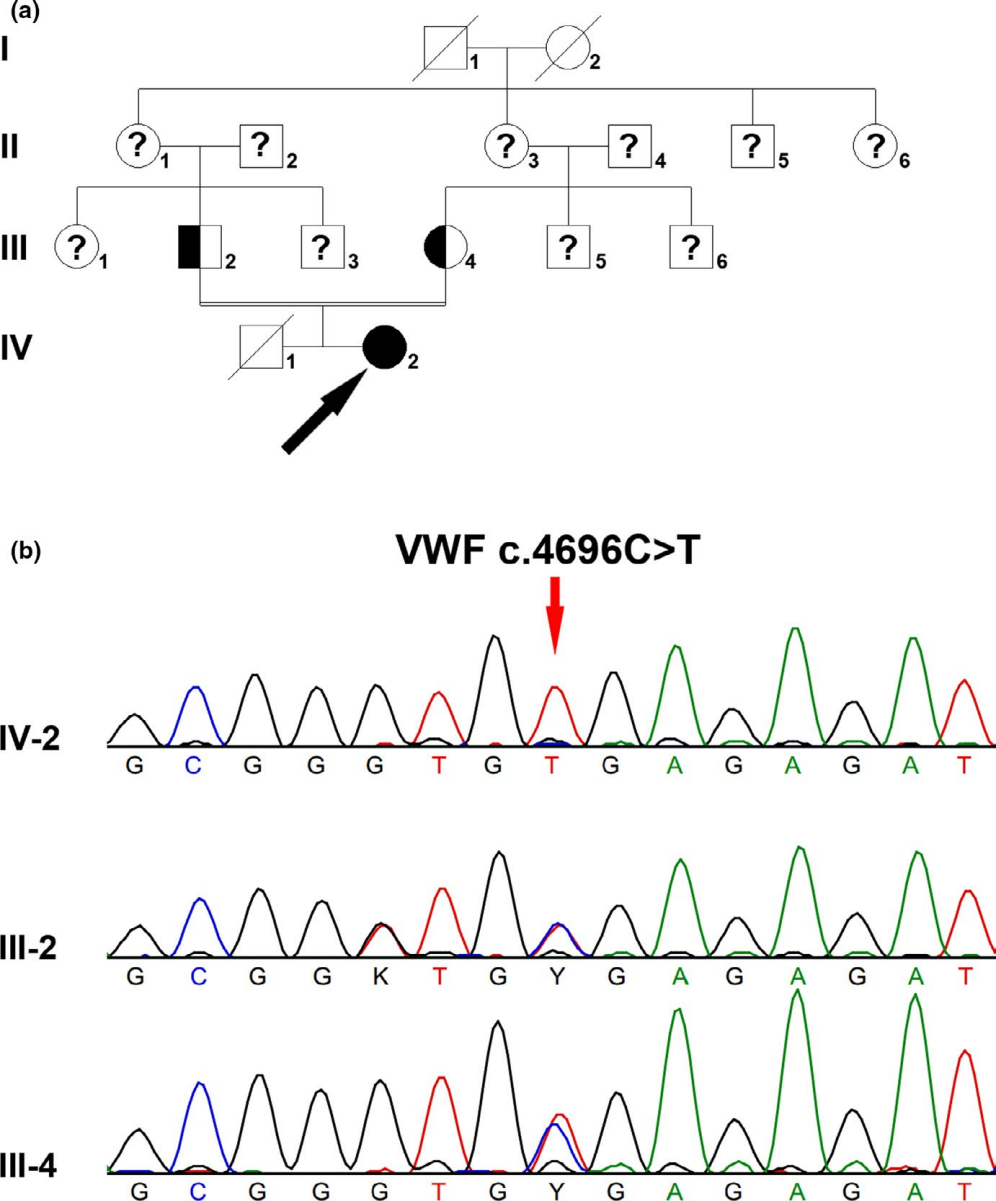
*VWF* c.4696C>T in VWD type 3. (a) Family tree of family A. Filled circle was patient, and half‐filled square or circle denoted carriers. Un‐filled square or circle represented unaffected male or female, respectively. An arrowhead denoted the proband. Question mark indicates the genetic test was not performed. (b) VWF c.4696C>T mutation in this family

A 23‐year‐old female patient suffered menorrhagia. When she was 2 years old, scalp hematoma was observed. Her prothrombin time (PT) and activated partial thromboplastin time (APTT) were both prolonged (PT: 15.9 s, APTT: 62.7 s), whereas thrombin time (TT) was normal (TT: 14.9 s). Both FVIII:C, VWF:Ag, and VWF:RCo were remarkably decreased (FVIII:C: 1.3%, VWF:Ag: 3.6%, VWF:RCo: <1.0%). The patient's parents were cousins, and this pair of parents had a boy who died when he was 1 year old due to heavy nose bleeding. The family tree is shown in Figure 1a.

Homozygous *VWF* c.4696C>T (p.Arg1566Ter) nonsense mutation was identified in the female patient, and both her parents were heterozygous carriers (Figure 1b). *VWF* c.4696C>T nonsense mutation was not found in either 1000G or ExAC database, whereas it was included in HGMD database(Kakela et al., 2006). The T allele of frequency was 0.000008142 in gnomAD database. No mutation was found in the *F8* or *F9* gene in the patient.

### 3.2 Family B

**Figure 2:**
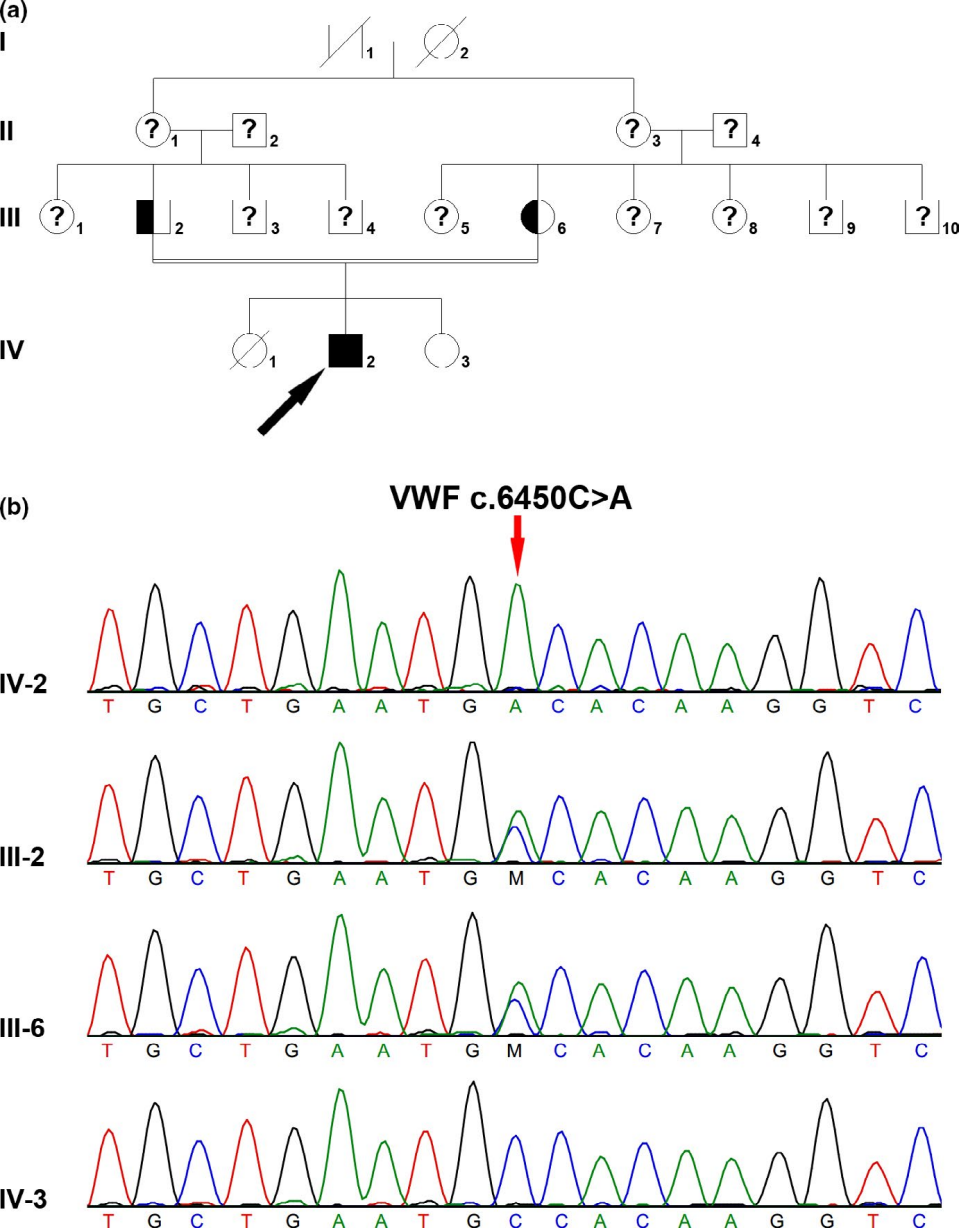
*VWF* c.6450C>A in VWD type 3. (a) Family tree of family A. Filled square was patient, and half‐filled square or circle denoted carriers. Un‐filled square or circle represented unaffected male or female, respectively. An arrowhead denoted the proband. Question mark indicates the genetic test was not performed. (b) VWF c.6450C>A mutation in this family

A 30‐year‐old male patient characterized with hematoma in the lower extremity, was referred to our hospital for treatment with recombinant Factor VIII. He had been misdiagnosed with hemophilia A for several years. The VWF:Ag, VWF:RCo, and FVIII:C were decreased to be 0.5%, 0.1%, and 1.2%, respectively. His PT and TT were within normal range, but APTT was prolonged (PT: 13.6 s, APTT: 72.0 s, TT: 14.9 s). The patient's parents were cousins, and this pair of parents had a son and two daughters (Figure 2a), and one daughter died when she was 1 year old due to bleeding disorder. Homozygous *VWF* c.6450C>A (p.Cys2150Ter) nonsense mutation was found in the patient, and both his parents were heterozygous carriers. The VWF:Ag and VWF:RCo of his mother with heterozygous mutation were 58% and 36%, respectively. His younger sister who showed normal VWF:RCo and VWF:Ag, did not carry this mutation (VWF:Ag: 103.7%, VWF:RCo: 110%). *VWF* c.6450C>A nonsense mutation was novel, and it was not found in 1000G, ExAC, gnomAD, or HGMD database. No mutation was found in the *F8* or *F9* gene in the patient.

## 4 DISCUSSION

von Willebrand factor serves as a carrier of FVIII to activate the coagulation cascade. Decreased circulating VWF level is associated with reduced FVIII. Reduced FVIII level is common to both VWD and HA. Moreover, in patients with VWD type 2N, the bleeding mimics the findings seen in classical HA. The finding of decreased FVIII level without consideration on VWF level and function may lead to misdiagnosis of VWD as HA (Favaloro & Lippi, 2018). In this study, the male patient in family B has been treated as HA for several years. Combined with VWF:Ag, VWF:RCo, and molecular test made the diagnosis of VWD type 3.

Molecular testing is indispensable for an accurate VWD subtype diagnosis and are particularly important for distinguish VWD 2N from HA. The VWD type 2N like HA only presents a decreased FVIII:C levels. NGS could analyze many genes simultaneously, and it has been routinely applied in clinical practice (Sivapalaratnam, Collins, & Gomez, 2017). Fidalgo et al., compared Sanger sequencing and NGS targeting *VWF* including promoter, exon, and the intronic flanking regions (at least 20 bp) (Fidalgo et al., 2016). Bastida et al., applied NGS approach for sequencing the *F8*, *F9*, and *VWF* genes. Their proposed algorithm had an overall success rate of 99% (Bastida et al., 2017). Moreover, Johnsen et al., had designed a novel NGS approach which could detect not only *F8* and *F9* sequencing, but also inversions of introns 1 and 22 simultaneously (Johnsen et al., 2017). Liang et al., combined NGS and CNVplex® technique to detect mutation and copy number of the *VWF* gene (Liang et al., 2017). The implementation of molecular study was suggested as the first‐line test for routine diagnosis of VWD (Borras et al., 2017). However, the presence of a highly homologous (>96% homology) pseudogene (exons 23–34) in chromosome 22 indicates that the molecular diagnosis of VWD should be treated with caution (Ahmad et al., 2013). The PCR may not distinguish *VWF* from its pseudogene due to the limited PCR product size designed by Ampliseq of Thermo Fisher platform or Amplicon of Illumina platform. The mutation identification may depend on raw data bioinformatic analysis via SNP and other features.

The proband of family B had been misdiagnosed with hemophilia A for several years probably due a wrong approach in first functional studies—the diagnosis was made based only in FVIII measurements. VWF analysis should be performed according to an algorithm based on established recommendations of SSC/VWD/ISTH: screening tests, specific tests, and genetic analysis (Castaman, Hillarp, & Goodeve, 2014). The functional studies in both families made the VWD diagnosis by FVIII:C; VWF:Ag and VWF:RCo values. However, the presence of only trace amounts of VWF and a FVIII:C below 5% could lead to the doubt between type 3 VWD and severe type 1 VWD. The molecular study found two nonsense variants, confirm type 3 VWD and allows the identification of carriers in these families, which are relevant and even mandatory for genetic counseling.

10.4% of marriages in the world have been estimated to occur between relatives (Bittles & Black, 2010). Consanguineous marriages may favor the increased frequency of AR diseases in a population (Fahiminiya et al., 2014; Li et al., 2017; Machado et al., 2013). Declining consanguinity would be reflected in decreased prevalence of complex diseases. In the current two families, VWD type 3 was caused by consanguineous marriage. Both *VWF* c.4696C>T and *VWF* c.6450C>A variants were very rare, and were not found in 1000G or ExAC database. *VWF* c.6450C>A variant has not been reported previously. In both families, they had a child who died due to bleeding disorders early in life, indicating that the two variants may cause type 3 VWD, the most severe VWD subtype. Our study further demonstrate that consanguineous marriage may increase the frequency of rare AR disease.

In conclusion, we identified VWD type 3 in two consanguineous marriage families, and our work further strengthen the risk of delivering disorders inherited in AR manner in populations with frequent consanguineous partnerships.

## CONFLICTS OF INTEREST

The authors declare that they have no competing interest.

## AUTHOR CONTRIBUTIONS

X.W., genetic tests; N.T., laboratory test; Y.L., Genetic counseling; Q.H., and D.L., design of this study.

## Data Availability

The data that support the findings of this study are available from the corresponding author upon reasonable request.
